# Accelerating imaging research at large-scale scientific facilities through scientific computing

**DOI:** 10.1107/S1600577524007239

**Published:** 2024-08-27

**Authors:** Chunpeng Wang, Xiaoyun Li, Rongzheng Wan, Jige Chen, Jing Ye, Ke Li, Aiguo Li, Renzhong Tai, Alessandro Sepe

**Affiliations:** a Big Data Science Center Shanghai Synchrotron Radiation Facility, Shanghai Advanced Research Institute, Chinese Academy of Sciences No. 239 Zhangheng Road Shanghai201210 People’s Republic of China; b Shanghai Synchrotron Radiation Facility, Shanghai Advanced Research Institute, Chinese Academy of Sciences No. 239 Zhangheng Road Shanghai201210 People’s Republic of China; NSRRC, Taiwan

**Keywords:** scientific computing, synchrotron, imaging, automation, tomography

## Abstract

To date, computed tomography experiments, carried-out at synchrotron radiation facilities worldwide, pose a tremendous challenge in terms of the breadth and complexity of the experimental datasets produced. Furthermore, near real-time three-dimensional reconstruction capabilities are becoming a crucial requirement in order to perform high-quality and result-informed synchrotron imaging experiments, where a large amount of data is collected and processed within a short time window. To address these challenges, we have developed and deployed a synchrotron computed tomography framework designed to automatically process online the experimental data from the synchrotron imaging beamlines, while leveraging the high-performance computing cluster capabilities to accelerate the real-time feedback to the users on their experimental results. We have, further, integrated it within a modern unified national authentication and data management framework, which we have developed and deployed, spanning the entire data lifecycle of a large-scale scientific facility. In this study, the overall architecture, functional modules and workflow design of our synchrotron computed tomography framework are presented in detail. Moreover, the successful integration of the imaging beamlines at the Shanghai Synchrotron Radiation Facility into our scientific computing framework is also detailed, which, ultimately, resulted in accelerating and fully automating their entire data processing pipelines. In fact, when compared with the original three-dimensional tomography reconstruction approaches, the implementation of our synchrotron computed tomography framework led to an acceleration in the experimental data processing capabilities, while maintaining a high level of integration with all the beamline processing software and systems.

## Abstract 2

Scientific computing augmentation paving the way for the advent of next-generation synchrotron imaging research is discussed.

## 1. Introduction

X-ray imaging methods are widely used to investigate the structural properties of materials at the macro-, micro- and nano-scale, as well as magnetic domain structures, spatial distribution of the elements, chemical properties, *etc.* (Xu *et al.*, 2016 ▸; Wang *et al.*, 2020 ▸; Zhang *et al.*, 2018 ▸, 2022 ▸; Suzuki *et al.*, 2018 ▸). The wavelength of X-rays is significantly shorter than that of the visible light, resulting in an imaging resolution several orders of magnitude higher than what it is possible to achieve with visible light. Furthermore, X-rays are characterized by possessing very high material penetration capabilities, thus enabling non-destructive imaging of the internal structures of thick samples. X-ray computed tomography (CT) is a non-destructive imaging technique that provides a clear, accurate and intuitive visualization of the internal structures, composition, properties and defects of the investigated materials, both through two-dimensional (2D) or three-dimensional (3D) image reconstructions, and it is widely recognized within the scientific community as one of the best non-destructive experimental methods (de Jonge *et al.*, 2010 ▸; Xing *et al.*, 2016 ▸). With the worldwide widespread use of third-generation synchrotron facilities, the number of opportunities to conduct cutting-edge scientific studies have been significantly increased. When compared with a traditional CT experiment, using X-ray laboratory sources, synchrotron CT (SR-CT) experiments provide remarkable advantages in terms of a better spatial resolution, improved temporal resolution and access to the multimodal X-ray imaging approach, when combined with X-ray spectroscopy and diffraction methods. Moreover, SR-CT is still undergoing a transformative development process, transitioning from 2D and 3D to multi-dimensional imaging, from morphology to functional imaging, from static to dynamic imaging, from single-scale to multi-scale investigations and from single-modal to multi-modal imaging (Xie *et al.*, 2019 ▸; Deng *et al.*, 2015 ▸; Hu *et al.*, 2017 ▸).

As an experimental technique that relies on digital raw data imaging, SR-CT benefits significantly from continuous innovation stemming from the field of X-ray detector technology, recently focused on increasing the number of pixels while reducing the pixel size. This enables the steady improvement of SR-CT spatial resolution capabilities, while increasing the amount of morphological details that it can capture. However, due to the substantial number (ranging from hundreds to thousands) of projections required by commonly used CT algorithms in order to generate the final reconstructions without the presence of significant artifacts, currently the size of a set of SR-CT datasets often exceeds several gigabytes (GB) of data. On the other hand, modern ultrafast imaging detectors allow for the continuous acquisition of data at rates exceeding 7.7 GB s^−1^ (Mokso *et al.*, 2017 ▸; García–Moreno *et al.*, 2021 ▸), achieving an unprecedented high temporal resolution, of more than 10 sets of SR-CT data throughput per second, while generating a series of continuous datasets representing the dynamic state of the sample. Consequently, SR-CT data processing becomes a computationally demanding task and a key critical issue for X-ray imaging experiments at synchrotron facilities worldwide. Therefore, the advancements brought by scientific computing at synchrotron facilities are of a crucial importance in order to support users to effectively evaluate and improve their dynamic CT experiments by utilizing full 3D reconstructed volumes, rather than previewing the original imaging projections or subsets of reconstructed slices, as the former contains considerably more information and detail about the current sample state. Furthermore, processing this large amount of data collected within a short period of time is crucial in order to achieve near-real-time 3D reconstruction capabilities (Wang *et al.*, 2018 ▸). This will then maximize the advantages of leveraging the potential of artificial intelligence (AI) technologies applied to experimental result-informed data processing and analysis.

Generally, depending on the adopted geometry, a high-quality CT data reconstruction workflow may involve numerous pre-processing and post-processing steps, including image normalization, geometric parameter correction, phase retrieval, filtering, smoothing, denoising and artifact removal. These steps involve the application of various image processing algorithms and parameter optimizations. Typically, these algorithms and parameters require iterative optimization and adjustment for each individual dataset, leading to a heavily manned interaction between the users and the reconstruction software, making the reconstruction quality highly dependent on the users’ level of experience and expertise. Therefore, the development of unmanned and fully automated high-performance CT data pipelines is both critical and a challenging obstacle to overcome. However, in the case of processing dynamic CT data and large-scale datasets from extended field of view or mosaic scan CT experiments (where large samples are scanned at different vertical and horizontal positions to obtain a set of datasets that are then stitched together to generate one large dataset) (Vescovi *et al.*, 2017 ▸; Borisova *et al.*, 2021 ▸), where the beamline status and experimental parameters can be regarded as static invariants, optimizations and adjustments of the algorithms and the parameters can be performed on a series of continuous experimental data flows. Therefore, the use of an automated processing pipeline can effectively maximize the experimental efficiency.

Currently, there are several open-source frameworks and toolkits for tomographic data reconstruction, as well as efforts aiming at building automatic, fast and flexible workflows based on them; for example, *Tomopy* (Gürsoy *et al.*, 2014 ▸; Pelt *et al.*, 2016 ▸), *TomocuPy* (Nikitin, 2023 ▸), *Tofu* (Faragó *et al.*, 2022 ▸), *Savu* (Atwood *et al.*, 2015 ▸; Wadeson & Basham, 2016 ▸; Kazantsev *et al.*, 2022 ▸), *UFO* (Vogelgesang *et al.*, 2012 ▸; Vogelgesang *et al.*, 2016 ▸), *SYRMEP Tomo Project* (Brun *et al.*, 2017 ▸), *SPOT* (Blair *et al.*, 2014 ▸; Deslippe *et al.*, 2014 ▸), *PyHST2* (Mirone *et al.*, 2014 ▸), *TOMCAT* post-processing pipeline (Marone *et al.*, 2017 ▸; Buurlage *et al.*, 2019 ▸), *etc*. (Hidayetoğlu *et al.*, 2020 ▸; Pandolfi *et al.*, 2018 ▸). They provide command-line interfaces (CLIs), graphical user interfaces (GUIs), or a combination of both, and achieve complex functionalities by offering auxiliary pre-processing and post-processing algorithms, as well as interfaces with other 3D reconstruction tools. They either use CPUs and GPU workstations or benefit from the computing power provided by national supercomputing centers in order to fully satisfy real-time reconstruction requirements. These software frameworks have been used for many years at many synchrotron large-scale facilities, whose development process demonstrates the importance of real-time, versatile, user-friendly and scalable reconstruction software in order to establish flexible and effective data processing workflows.

In this study, a software framework, architected, developed and deployed by the Big Data Science Center (BDSC) at the Shanghai Synchrotron Radiation Facility (SSRF), and aiming at providing online automated processing and real-time feedback on the experimental data produced by the synchrotron imaging beamlines at the SSRF, is presented (Sepe *et al.*, 2024 ▸). This framework leverages the resources and capabilities provided by the BDSC (Wang *et al.*, 2021 ▸; Ye *et al.*, 2023 ▸), including its high-performance computing (HPC) systems, unified authentication and user management system, Big Data and metadata framework, as well as its AI-SSRF-Superfacility Platform (AI-SSRF-SP), to accelerate and automate the scientific data analysis and processing pipelines, while fully integrating them into the imaging beamlines, thereby enhancing their overall scientific productivity.

The remainder of this article is organized as follows. In Section 2, we introduce the imaging beamlines at the SSRF and provide an overview on the various infrastructures and existing capabilities provided by the BDSC to the SR-CT community; Section 3 presents the detailed implementation of the software framework developed and deployed by the BDSC to accelerate and augment the SR-CT experiments; Section 4 provides an overview on the experimental performance improvements as a result of the deployment of the BDSC CT scientific computing framework, and illustrates its impact, presenting scientific case studies involving the SR-CT experiments accelerated and augmented by the BDSC CT scientific computing framework; finally, in Section 5 the conclusions and future prospects are presented.

## 2. The SSRF and the Big Data Science Center

### 2.1. The SSRF and the SSRF Phase-II Beamline Project

The SSRF is the first medium-energy third-generation synchrotron light source on the Chinese mainland, featuring a 150 MeV linear accelerator, a 3.5 GeV booster, a 3.5 GeV storage ring, a circumference of 432 m and an emittance of about 4 nm rad (Jiang *et al.*, 2009 ▸; Yin *et al.*, 2016 ▸). The SSRF was established and became operational in 2009. Since then, it has supported nearly 700 research institutes, universities and industries worldwide, with a cumulative user base exceeding 37000 users. The SSRF Phase-II Beamline Project is one of the key national scientific infrastructure projects of the 12th five-year plan period, which was launched in 2016 and was fully completed in July 2023 (Tai & Zhao, 2022 ▸). The main goal of the SSRF Phase-II Beamline Project is to significantly improve the performance and capabilities of the SSRF, and to meet the growing needs of the modern science and technology (Tai & Zhao, 2022 ▸). This project comprises 18 novel beamlines and multiple endstations, accelerator upgrades, user supporting laboratories, including the BDSC, the beamline engineering and technology laboratories, as well as utilities and buildings, housing the BDSC, the user supporting facility and the user training facility.

To date, the SSRF has 34 beamlines and 46 endstations in operation (Fig. S1 of the supporting information). Having completed the Phase-II Beamline Project update, the SSRF is now able to provide comprehensive user support, including massive data storage, HPC systems, cloud/edge hybrid frameworks and the AI-assisted Big Data analysis and interpretation superfacility platform (Wang *et al.*, 2021 ▸).

### 2.2. The Big Data Science Center

To address the Big Data deluge challenges at the large scientific facilities, the SSRF has strategically planned the BDSC as a component of the SSRF Phase-II Beamline Project. The BDSC is founded on the concept of implementing a superfacility at the SSRF (Wang *et al.*, 2021 ▸). Therefore, the BDSC has developed and deployed a synchrotron big data framework that comprehensively integrates, within the SSRF, a large scientific facility experimental infrastructure with the HPC and supercomputing resources, scientific edge/cloud infrastructure, the Internet of Things (IoT) and AI capabilities, virtualization framework, low-latency high-throughput network, remote access infrastructure, the most updated theoretical science framework, producing the most advanced algorithms for science, and a user superfacility platform, enhancing their scientific productivity. This framework centralizes, unifies, manages, curates and formats the users’ data from all the beamlines throughout the entire SSRF, enabling a comprehensive facility-wise data lifecycle management. At the same time, it provides a high-performance big data analysis and processing framework to the SSRF users and beamlines, facilitating the integration and centralization of all the experimental data pipelines, while fostering the real-time analytical capabilities at the SSRF.

To date, the BDSC metadata system has cataloged more than 47000 datasets, exceeding 1.6 PB, resulting from over 1200 SSRF user research proposals. The beamlines at the SSRF have thus highly benefitted from the BDSC scientific computing architecture, as demonstrated by the integration of the Aqua­rium pipeline (Yu *et al.*, 2019 ▸) from the macromolecular crystallography beamline (BL02U1) and the Biosafety P2 protein crystallography beamline (BL10U2) into the BDSC scientific computing framework, which resulted in a significant improvement of their on-line data processing capabilities (Wang *et al.*, 2021 ▸).

### 2.3. The imaging beamlines

SSRF currently has several imaging beamlines available to its users, including the X-ray imaging and biological application beamline (BL13HB) (Xie *et al.*, 2015 ▸), the 3D nano imaging beamline (BL18B) (Tao *et al.*, 2023 ▸) and the fast X-ray imaging beamline (BL16U2) (Tai & Zhao, 2022 ▸). The X-ray imaging experimental methods include X-ray micro-CT, dynamic micro-CT, X-ray nano-CT, X-ray fluorescence computed tomography (XFCT), small-angle X-ray scattering (SAXS)-CT, full-field transmission X-ray microscopy (TXM), *etc.* (Xie *et al.*, 2020 ▸). The fast X-ray imaging beamline is dedicated to both fast X-ray imaging and dynamic X-ray micro-CT, providing experimental temporal imaging resolution from milliseconds, to microseconds, to ∼100 ps. During commissioning, it has also successfully performed single-shot synchrotron X-ray imaging with 60 ps time resolution (X-ray pulse duration time) in a hybrid bunch filling mode with a maximum single bunch up to 23 mA (Tai & Zhao, 2022 ▸). The 3D nano imaging beamline is dedicated to full-field transmission X-ray microscopy and full-field X-ray nano-tomography.

## 3. Integrating the imaging beamlines

### 3.1. Imaging data pipelines

**Figure 1:**
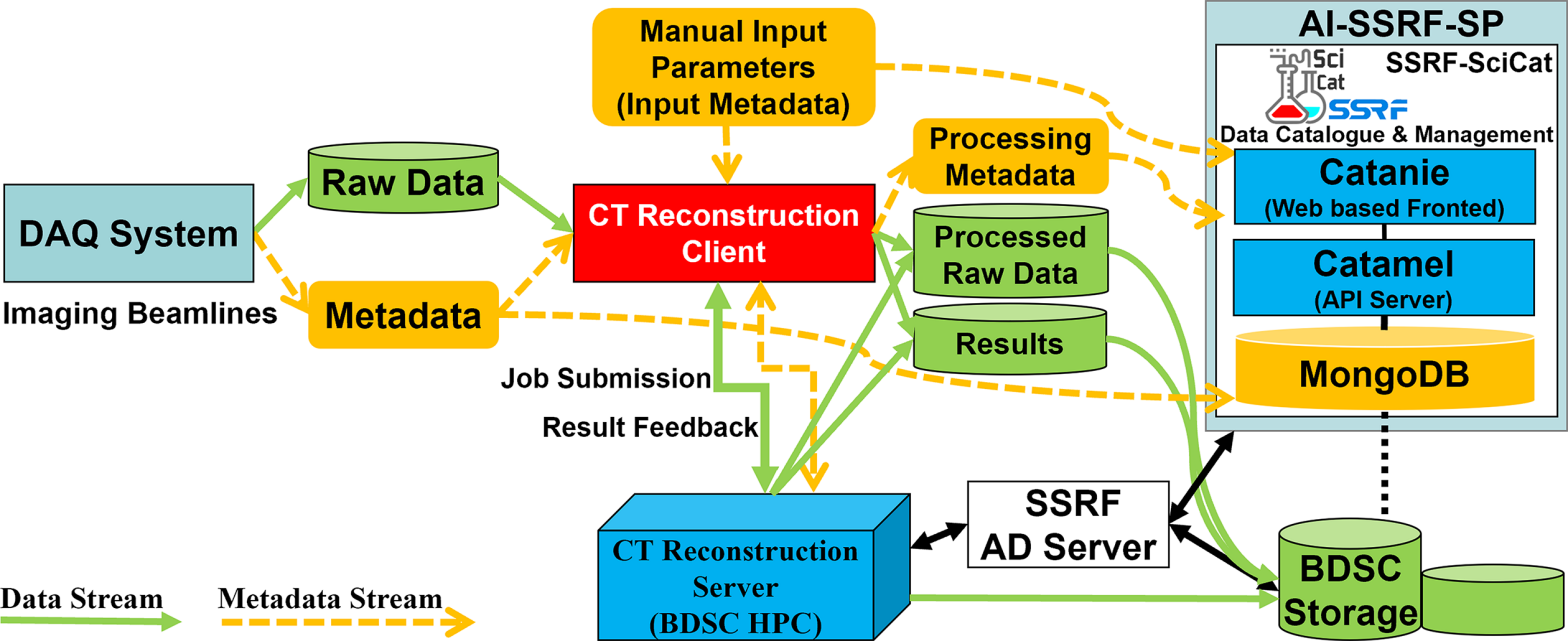
Design of the SR-CT pipeline system.

The overall design target for the BDSC, developing the SR-CT pipeline system, is to deploy and provide a software framework capable of real-time automatic experimental data processing, analysis and result feedback to all users at SSRF imaging beamlines, as shown in Fig. 1 ▸ (Sepe *et al.*, 2024 ▸). Architected, developed and deployed over a period of one year, this framework aims at fully utilizing the HPC resources and capabilities provided by the BDSC, including its unified authentication management system, its metadata management system (SSRF-SciCat) and its AI-SSRF-SP, to accelerate and automate the SR-CT experimental data analysis pipeline at the imaging beamlines, thereby enhancing the users’ scientific productivity. Furthermore, the BDSC staff’s modular and multidisciplinary skill sets are leveraged, encompassing a portfolio of expertise from scientific computing to material science, bioscience, synchrotron science, data science, computational science, chemistry, physics, and software and hardware engineering. The BDSC SR-CT software framework is capable of running the SR-CT reconstruction applications on the BDSC HPC cluster systems, effectively utilizing the CPU and GPU computing resources, supporting the massively parallelized CT reconstruction computing tasks, thereby significantly reducing the online data processing time. Furthermore, it provides fully automatic SR-CT experimental data processing pipelines, equipped with a GUI and services tightly integrated within the BDSC, while adopting an advanced architecture that is efficient, stable, reliable and scalable. These pipelines provide real-time feedback and monitoring of the pipeline task status. The BDSC SR-CT framework fully utilizes the SSRF-SciCat metadata management system, linking all the data and input/output parameters resulting from the SR-CT reconstruction applications with the corresponding experimental data and metadata. All the scientific metadata are then aggregated into a JavaScript Object Notation (JSON) universal file format, which are then accessed by the AI-SSRF-SP platform with the purpose of training the AI through machine learning (ML). Moreover, it implements a unified and standardized full data lifecycle management for the raw data, processed data and results produced by the users’ experiments at the imaging beamlines. It also provides to the users a fully integrated solution for all the data services accessible through the AI-SSRF-SP. Additionally, it does integrate the BDSC unified authentication system into the SR-CT experimental pipelines, providing a robust authentication management system ensuring overall data privacy and security.

### 3.2. Integration of the SR-CT reconstruction applications

The integrated SR-CT reconstruction application is a specialized 3D CT reconstruction software developed and tailored on the specific data workflow used by the X-ray nano- and micro-CT beamlines, and it is designed to seamlessly operate within an HPC cluster environment. The application consists of two components: a GUI, tailored for the Windows operating system, and a server-side module. The GUI allows users to import data, to use sample-specific features for image correction and alignment, and to set pre-reconstruction parameters using a graphical interface, which, further, allows users to seamlessly submit CT reconstruction computational tasks, for processing, to the BDSC HPC clusters. The server-side component, instead, is in charge of running the high-performance 3D reconstructions, based on the parameters provided by the users through the GUI (Sepe *et al.*, 2024 ▸).

The reconstruction application functionalities provide comprehensive support for the full-field nanoscale CT and microscale CT scanning data reconstructions, while supporting the selection of the reconstruction algorithms, including the filtered back-projection (FBP) and the algebraic reconstruction technique (ART). They integrate the ordered subset expectation maximization (OSEM) algorithm, and the capability of performing missing angle reconstruction. Furthermore, they provide GPU-accelerated real-time and efficient 3D reconstruction capabilities, and support for image pre-processing capabilities (background subtraction, filtering, smoothing, *etc*.), for ring artifact removal, for the batch reconstruction of the micro-CT data and for semi-automatic geometric parameter correction.

Meanwhile, the application GUI functionalities include a client–server architecture, designed to operate within a LAN, and facilitating seamless client–server interactions. Furthermore, they support manual annotation and correction driven by sample-specific features inside the nano-CT projection data; pre-reconstruction of the projection data, allowing a quick preview of the reconstruction results; region of interest reconstructions, enabling the users to select the desired reconstruction area; sample spatial orientation correction, allowing the users to adjust the sample spatial orientation as-needed; and real-time 3D multi-planar reformation (MPR) rendering of the reconstructed data.

### 3.3. Framework architecture

**Figure 2:**
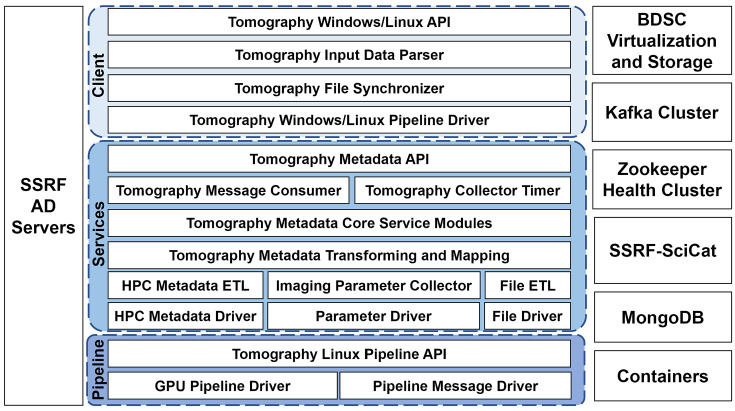
Architecture of the SR-CT framework.

The architecture of the SR-CT pipeline framework is shown in Fig. 2 ▸ (Sepe *et al.*, 2024 ▸). The framework comprises several components: the SSRF-SciCat scientific metadata management system that has been deployed by the BDSC; the Zookeeper node governance cluster; the Kafka message management cluster; the SSRF Active Directory (AD) domain account authentication system; and the BDSC HPC storage. The overall architecture consists of three layers: the system driver layer; the core framework service layer; and the client application layer, as shown in Fig. 2 ▸ (bottom-up).

The system driver layer, which includes the GPU pipeline driver, the pipeline message driver and the Linux Application Programming Interface (API) for the tomography pipeline, runs on the BDSC cluster and is responsible for providing a standard API interface for the GPU task submission pipeline and scheduling management, and for sending task-end messages to the Kafka message management cluster, once a task ends.

The core framework service layer runs on the BDSC data processing node, and it is responsible for receiving and processing pipeline task status messages, initializing the imaging beamline data collection and processing programs, parsing and extracting all the input and output metadata, in real-time, from the imaging beamline reconstruction pipelines, as well as for parsing the metadata from the scheduling system. Based on the management rules set for the SSRF-SciCat scientific metadata system, all the extracted metadata are then persistently stored within the SSRF-SciCat metadata repository.

The client application layer runs on the terminal workstations at the imaging beamlines, and it is responsible for providing the processing pipeline APIs to the integrated SR-CT reconstruction applications. Both the client and the reconstruction applications are deployed together. When the reconstruction applications utilize the BDSC HPC resources for the reconstructions, then the pipeline client is launched, accordingly. It receives input and data from the reconstruction applications, parses the specified parameter files, submits the computational tasks to the BDSC and then returns the status of BDSC data processing and the path to the output data. The reconstructed data can, then, be accessed directly from the imaging beamline workstations. The client application layer supports two operating modes: a Windows client mode and a Linux client mode, with a unified account and storage management system provided for both Windows and Linux systems.

### 3.4. Functional modules

The various modules developed by the BDSC for the SR-CT framework (Sepe *et al.*, 2024 ▸), along with their respective categories, specific functionalities and deployment locations, can be found in Table S1 of the supporting information.

### 3.5. Framework workflow

**Figure 3:**
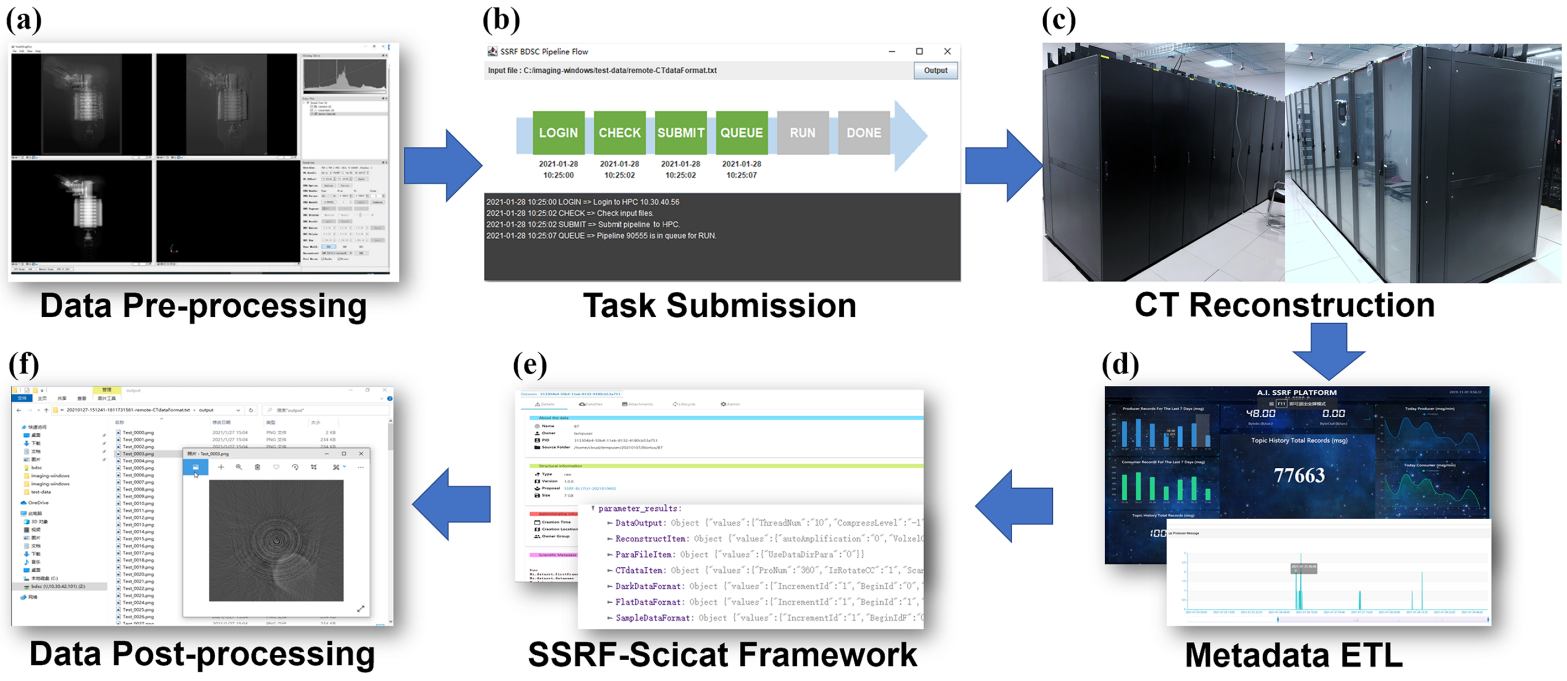
Workflow of the SR-CT pipeline framework.

The complete workflow of the SR-CT pipeline framework (Sepe *et al.*, 2024 ▸) is shown in Fig. 3 ▸. Users log in to the imaging beamline workstation using their SSRF AD credentials. The SR-CT reconstruction application is then launched on the beamline workstation. The users’ input files are upload to the workstation and the parameters for the data reconstruction are configured [Fig. 3 ▸(*a*)]. The pipeline task is then submitted, with the SR-CT reconstruction application invoking the BDSC pipeline client in order to submit the task to the remote BDSC HPC clusters [Fig. 3 ▸(*b*)]. When the reconstruction task is completed, the message bus is triggered [Fig. 3 ▸(*c*)]. The imaging beamline data processing service then receives a message and retrieves the task record and the metadata files from disk [Fig. 3 ▸(*d*)]. Subsequently, the imaging beamline data processing service parses the metadata and ingests them into the SSRF-SciCat system [Fig. 3 ▸(*e*)]. The beamline workstation then mounts the shared storage, and the users can see the 3D reconstruction results directly on the beamline workstation [Fig. 3 ▸(*f*)].

### 3.6. Data transfer and management

The BDSC scientific computing architecture enables the imaging beamline workstation, which is integrated within the SSRF AD domain, to mount and access the BDSC net­work storage (Sepe *et al.*, 2024 ▸). Using the SR-CT reconstruction application, the SSRF users can, thus, select the reconstruction input data from either the imaging beamline local workstation storage or the BDSC network storage.

When the input data are located on the local workstation storage, the BDSC SR-CT framework automatically detects and synchronizes them with the reconstruction task directory under the current AD account path on the BDSC. Each reconstruction task directory is named by default after the input file name and a timestamp. On the other hand, when the input data are stored on the BDSC network storage, the BDSC SR-CT framework does not synchronize the data. Instead, it generates a parameter file anew, and updates the parameter path with the actual storage location. The reconstructed data, generated by the reconstruction task, will be then stored inside the output directory, which is located inside the corresponding reconstruction task directory. Users can then directly access their data from the output directory at the beamline workstation.

### 3.7. Metadata

**Figure 4:**
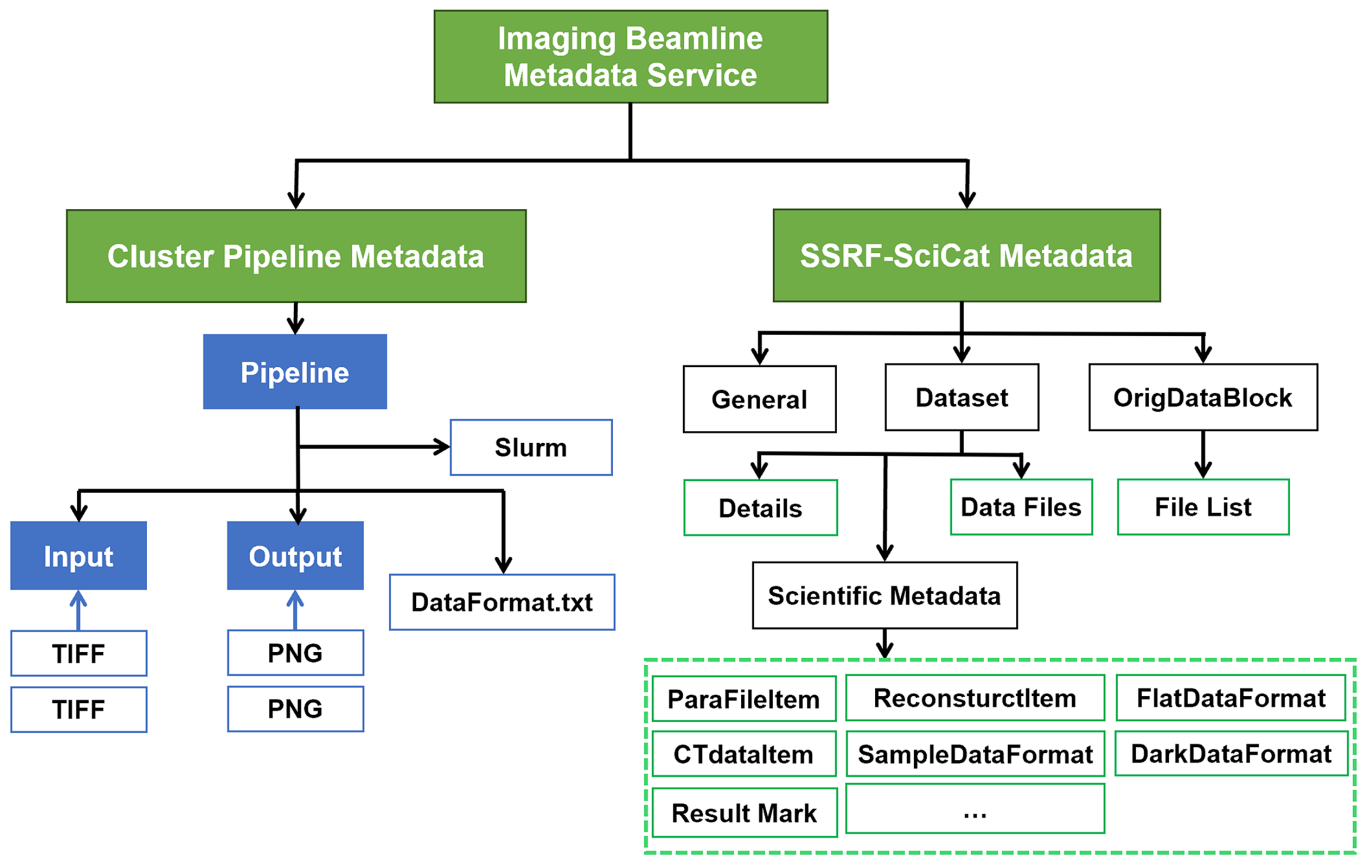
Metadata architecture of the imaging pipelines.

As illustrated in Fig. 4 ▸, the imaging beamline metadata service parses and extracts all the input and output metadata during the reconstruction pipeline tasks; it also extracts the metadata from the scheduling system, and then feeds all the extracted metadata to the SSRF-SciCat (Sepe *et al.*, 2024 ▸). Within the SSRF-SciCat metadata structure, all the most relevant and crucial experimental information are tagged with the #Scientific_metadata label, the #dataset label tags all the information related to the data files, while the #General label tags the additional metadata from the SSRF user proposal management system and from the unified account authentication service (Wang *et al.*, 2021 ▸).

### 3.8. GUI

During the SR-CT pipeline execution, a GUI is automatically initiated and made available to the users, further providing an intuitive overview on the pipeline progress to the users (Sepe *et al.*, 2024 ▸). The GUI does highlight each step within the pipeline execution progress, using colored boxes and arrows (Fig. S2), where blinking green highlights the step that is currently executed, solid green highlights that the step has been completed, and solid gray highlights that the step is queued.

The pipeline execution procedure is divided into six stages, as illustrated in Fig. S2 of the supporting information, where, from left to right, LOGIN reports the user’s login status, CHECK reports the framework input file check status, SUBMIT reports the computing job submission status, QUEUE reports the computing job queuing status, RUN reports the computing job execution status, and DONE reports the accomplishment status of the entire pipeline.

### 3.9. Framework benefits

The BDSC SR-CT framework provides several advantages and improvements to the imaging experiments at the synchrotron facilities:

(i) *Application-agnostic.* The system is highly decoupled from the SR-CT reconstruction applications and other systems at the imaging beamline. It is not limited to a specific imaging application, and it can easily integrate a large plethora of different CT reconstruction software. Moreover, only very minimal modifications are required in order to create a completely new automated processing pipeline for a totally different imaging application or task.

(ii) *Metadata management.* With the deployment of the SR-CT framework, the BDSC established a standardized tagging system for the labeling of the synchrotron imaging metadata parameters. It can automatically parse the parameter and value tags, and then submit tasks and synchronize data, accordingly. Other CT reconstruction applications can, thus, output parameters based on the BDSC SR-CT tagging system specifications. Moreover, the BDSC SR-CT framework is also capable of parsing customized parameter and value tags from other systems and different applications, and convert them into a standard metadata structure, ultimately feeding them into the SSRF-SciCat system.

(iii) *SR-CT beamline reconstruction software integration.* The framework client is developed using the Java programming language, providing a cross-platform user interface, while being able of integrating other software developed using different programming languages. Real-time data processing and analysis is achieved through inter-process communication via the deployment of process pipelines. This allows seamless integration with any software which could be developed in the future using the same communication method.

(iv) *High-performance task processing.* The BDSC SR-CT framework integrates the BDSC cluster task submission system and the query interfaces on the server-side, using a cross-platform REpresentational State Transfer (REST) API. The BDSC dynamically schedules tasks based on the resource availability, eliminating the need for the clients to be tightly bound to a specific set of resources, thus fostering scalability and the automatic allocation of the processing resources.

## 4. Results

### 4.1. Improvements of the imaging experimental performances

To assess the performance improvements brought by the SR-CT pipeline framework to the imaging experiments, we conducted performance evaluations in a production environment using the fast X-ray imaging beamline (BL16U2) workstations and a series of dynamic CT experimental datasets. Each set of the dynamic CT raw data consists of 250 (2000 × 1007 pixels) projections, with each projection being 3.84 MB in size. The total size of a single set of the dynamic CT raw data is 960 MB. For each dataset, the reconstructed (2000 × 2000 × 1007 voxels) 3D result data are stored in .RAW files, 7.5 GB in size. Then, these .RAW files can be split into 1007 (2000 × 2000 pixels) slices using .TIFF format, with each slice being 7.63 MB in size.

We carried out 3D reconstructions, both on individual datasets and on a series of datasets, using both the *PITRE* reconstruction program (Chen *et al.*, 2012 ▸), which utilizes the computing resources of the local workstation, specifically an Intel Xeon Gold 6244 CPU, comprising eight cores operating at 3.6 GHz, equipped with 256 GB of RAM and running the Microsoft Windows 10 operating system, and the BDSC centralized SR-CT pipeline framework, which is running the CentOS Linux 7 operating system. For batch parallel reconstructions, we utilized four GPU nodes on the BDSC clusters, with eight threads, where each node was equipped with two Intel Xeon 5118 CPUs, with 12 cores at 2.3 GHz, 128 GB of RAM and two NVIDIA Tesla P100 GPU cards. In both approaches, the FBP algorithm was used for the reconstructions. The results, as shown in Table 1 ▸, demonstrate that, compared with the original *PITRE* software used at the SSRF imaging beamlines, the 3D reconstructions for a single dataset, using the BDSC SR-CT pipeline, are accelerated by a factor of 7.84, while, in the case of the batch 3D reconstructions, they are accelerated by a factor of up to 59.34. With regard to the BDSC SR-CT pipeline, the typical time from task submission to the beginning of queuing is approximately 5 s, with an additional 30 s of queuing time before running on the node, and with a total completion time of approximately 11 min. The acceleration hereby introduced is thus resulting from accurately hybridizing the BDSC Big Data framework with its HPC infrastructure. In particular, the BDSC Big Data framework capabilities, which are designed to centralize, unify, manage, curate and format the experimental data from the beamlines, can be leveraged to transition the raw data into a metadata format that is ingested by the advanced data analysis, reconstruction and visualization, as well as machine learning, pipelines, which are fully integrated within the BDSC HPC cloud/edge underlying hybrid infrastructure.

### 4.2. Case studies

To showcase the impact of the BDSC SR-CT framework on the experimental studies employing the SR-CT, case studies, from the typical application area of the SR-CT research, are presented employing the BDSC SR-CT framework.

**Figure 5:**
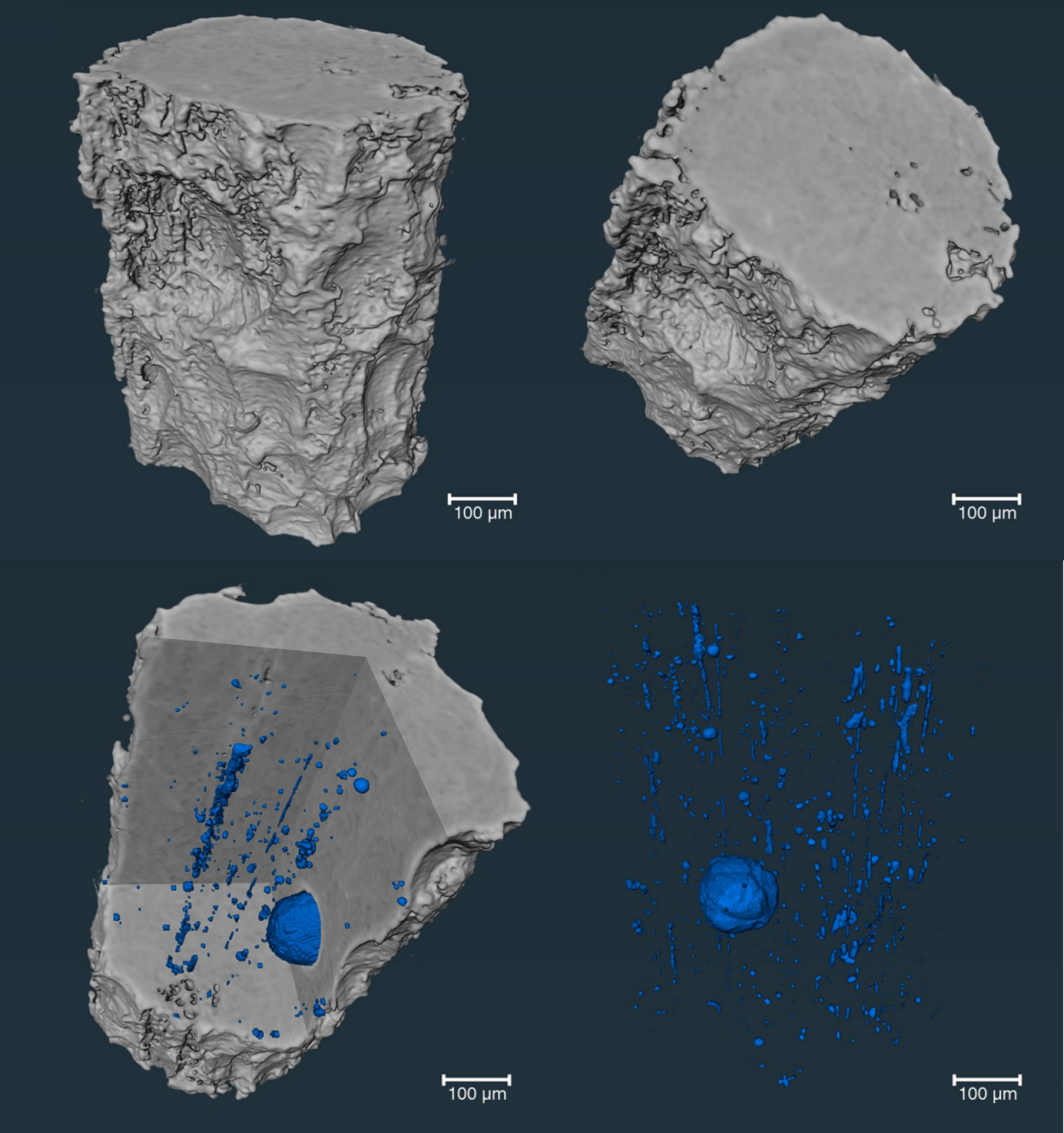
3D imaging of the pores and defects in the magnesium–aluminium alloy. (*a*) 3D reconstruction image, (*b*) cross-sectional view through the largest pore present in the alloy, (*c*) selected region of interest for slicing within the 3D reconstructed volume, and (*d*) corresponding longitudinal view of the 3D distribution and structure of the internal pores, are shown.

Fig. 5 ▸ shows the experimental results from the SR-CT 3D imaging analysis, at the X-ray imaging and biological application beamline (BL13HB), of the pores and defects within a micro-fabricated magnesium–aluminium (Mg–Al) alloy. Fig. 5 ▸(*a*) shows the reconstructed 3D image, while Fig. 5 ▸(*b*) illustrates a cross-sectional view through the largest pore present in the alloy. Figs. 5 ▸(*c*) and 5 ▸(*d*), instead, show a selected region of interest for slicing within the 3D reconstructed volume and the corresponding longitudinal view of the 3D distribution and structure of the internal pores, respectively. The scale bar in the figures represents 100 µm. The smallest pore present in the alloy measures, approximately, a few tens of micrometres. By employing the 3D imaging technique, the spatial distribution of these features can be analyzed in order to assess the impact of the manufacturing process on the samples and evaluate their qualities. This case study illustrates the acceleration achieved by implementing the BDSC centralized SR-CT pipeline on the SSRF imaging beamlines, with a factor of 10.37 increase in experimental analysis speed (Table 1 ▸).

**Figure 6:**
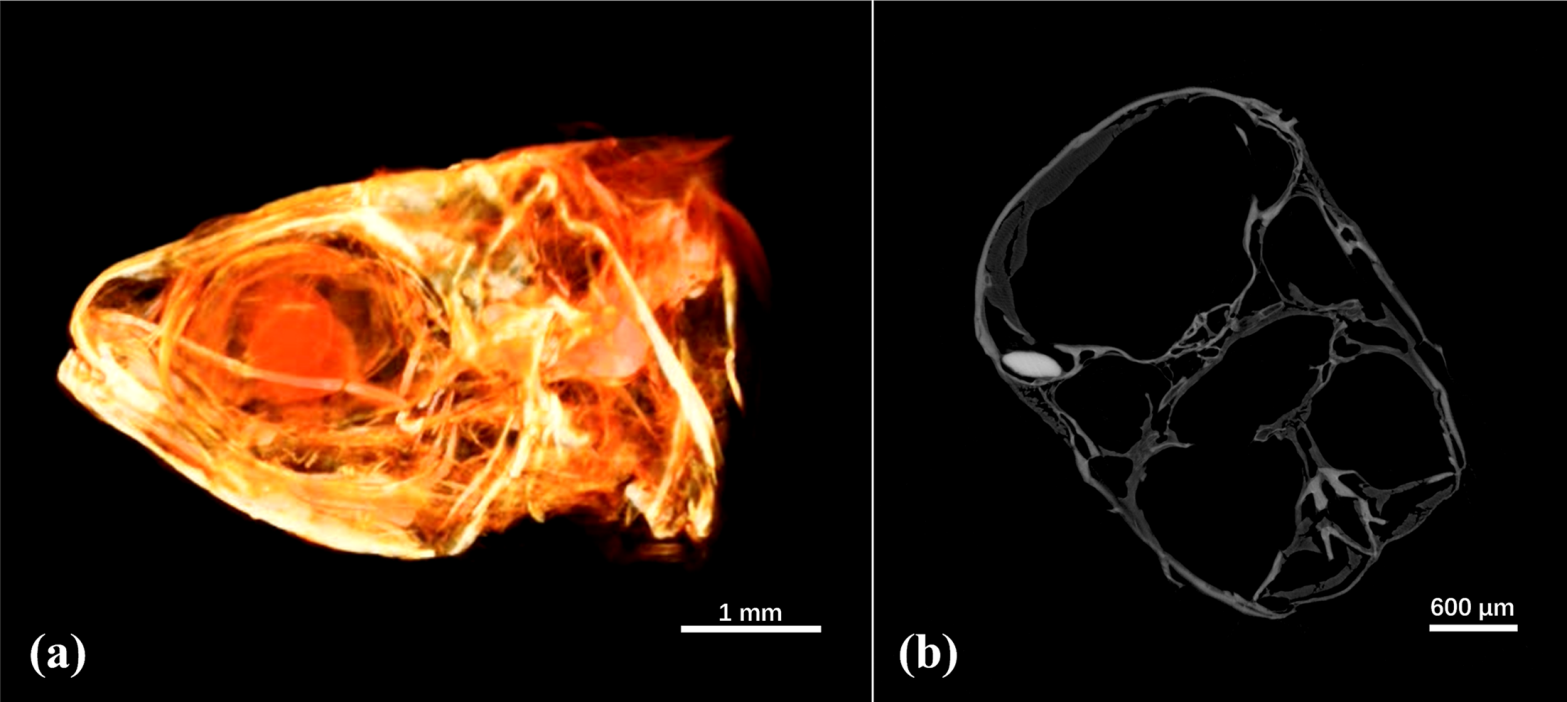
SR-CT (*a*) 3D phase-contrast imaging, and (*b*) corresponding frontal 2D reconstructed slice, of a fish head (*Poecilia reticulata*), where the different biological tissue structures can be clearly discriminated.

The experimental result presented in Fig. 6 ▸ shows SR-CT phase-contrast imaging, and the corresponding frontal 2D reconstructed slice, of a fish head (*Poecilia reticulata*), where a 3D reconstruction is obtained after phase retrieval, leveraging the high spatial resolution (0.325 µm pixel^−1^) provided by the X-ray micro-CT beamline. As shown in the figure, despite the intricate structure of the fish head, the 3D imaging technique enables the discrimination of its various biological tissue structures. The scale bar in Fig. 6 ▸(*a*) represents 1 mm, with the smallest feature present in the fish head measuring, approximately, a few tens of micrometres. 3D high spatial resolution phase contrast SR-CT allows observation of the tiniest structural details and features in bones, blood vessels, nerve fibers, *etc*., which is crucial for the investigation of the spatial distribution, morphological changes, internal structures, *etc*., occurring in complex biological structures, thus allowing for a more in-depth comprehensive study. This case study provides further evidence of the acceleration achieved through the implementation of the BDSC centralized SR-CT pipeline on the SSRF imaging beamlines, with a factor of 9.5 increase in experimental analysis speed (Table 1 ▸). Movie S1 illustrates the imaging results.

## 5. Conclusions

The BDSC has architected, developed and deployed, at the SSRF, an SR-CT pipeline software framework capable of effectively harnessing the BDSC scientific computing resources in order to accelerate and augment large-scale, massively parallelized, 3D CT experimental reconstructions at the imaging beamlines. The imaging beamlines at SSRF have been, in fact, fully integrated within the BDSC scientific computing framework, thus enabling the full automation of the real-time data processing and analysis pipelines and feedback, significantly reducing the time necessary for the users to process and analyze their experimental data. Furthermore, the BDSC has also integrated its SR-CT framework into the SSRF unified authentication management system (which has also been developed by the BDSC), thus enhancing the level of the framework data privacy and security. Moreover, the BDSC has architected and deployed the SR-CT metadata infrastructure at the SSRF, where all the inputs and outputs, including data and parameters, are ingested by the SSRF-SciCat system, and aggregated into the JSON universal file format, thus enabling the training of scientific AI through ML approaches. The limitations presented by the *PITRE* software, which lacked crucial HPC features, including the lack of parallelization capabilities and a codebase relying heavily on CPUs rather than being optimized for harnessing GPU architectural acceleration, proved to be a significant challenge for the BDSC during the design and integration phases of the imaging experimental pipeline into the BDSC HPC infrastructure. To address this limitation, the BDSC has thus designed, developed and deployed a framework capable of wrapping the *PITRE* software with a software layer, equipped with a GPU-aware scheduler and massive parallelization capabilities. This framework is able to translate non-HPC routines into HPC-optimized reconstructions, effectively allowing scientific software, not optimized for the HPC architecture, to access all the benefits of a modern scientific computing framework. The performance evaluation of the BDSC SR-CT framework demonstrates the extent of the acceleration induced by the SR-CT experimental data processing and analysis capabilities compared with the traditional beamline CT reconstruction approaches, impacting the application areas of the synchrotron imaging methods. The BDSC SR-CT framework architecture is highly decoupled from the beamline data infrastructure and the other systems; this allows it to be generalizable, adaptable, scalable, application agnostic and modularly expandable, thus facilitating its integration into the most diverse SR-CT software frameworks addressing the most heterogeneous scientific cases at other facilities worldwide, while seamlessly and quickly adapting to the new challenges posed by the evolution of the future SR-CT experiments, without requiring a structural change in its architecture.

## Supplementary Material

Figure S1: SSRF beamline layout; Table S1: functional modules of the SR-CT framework; Figure S2: the UI of the SR-CT pipeline framework. DOI: 10.1107/S1600577524007239/ju5063sup1.pdf

Imaging results. DOI: 10.1107/S1600577524007239/ju5063sup2.mp4

## Figures and Tables

**Table 1:** Results from assessment of the acceleration brought by the BDSC centralized SR-CT pipeline to the synchrotron imaging experimental performances, when compared with the *PITRE* suite, using different numbers of CT datasets and samples

CT datasets	*PITRE*	SR-CT pipeline	Acceleration factor
1	90.95 min	11.6 min	7.84
8	727 min	12.25 min	59.34
12	1091 min	23.65 min	46.13
Mg–Al alloy	197 min	19 min	10.37
*Poecilia reticulata*	172 min	18.1 min	9.5

## Data Availability

The authors confirm that the data supporting the findings of this study are available within the article and its supplementary materials.
