# Supplementary material for: Accelerating imaging research at large-scale scientific facilities through scientific computing
Source: J Synchrotron Radiat. 2024 Aug 27;31(Pt 5):1317–26. doi: 10.1107/S1600577524007239 (PMC11371030; doi:10.1107/S1600577524007239)
Supplement: Supplementary file 1 [file s-31-01317-sup1.pdf]

JOURNAL OF SYNCHROTRON RADIATION

Volume 31 (2024)

Supporting information for article:

Accelerating imaging research at large-scale scientific facilities through scientific computing

Chunpeng Wang, Xiaoyun Li, Rongzheng Wan, Jige Chen, Jing Ye, Ke Li, Aiguo Li, Renzhong Tai and Alessandro Sepe

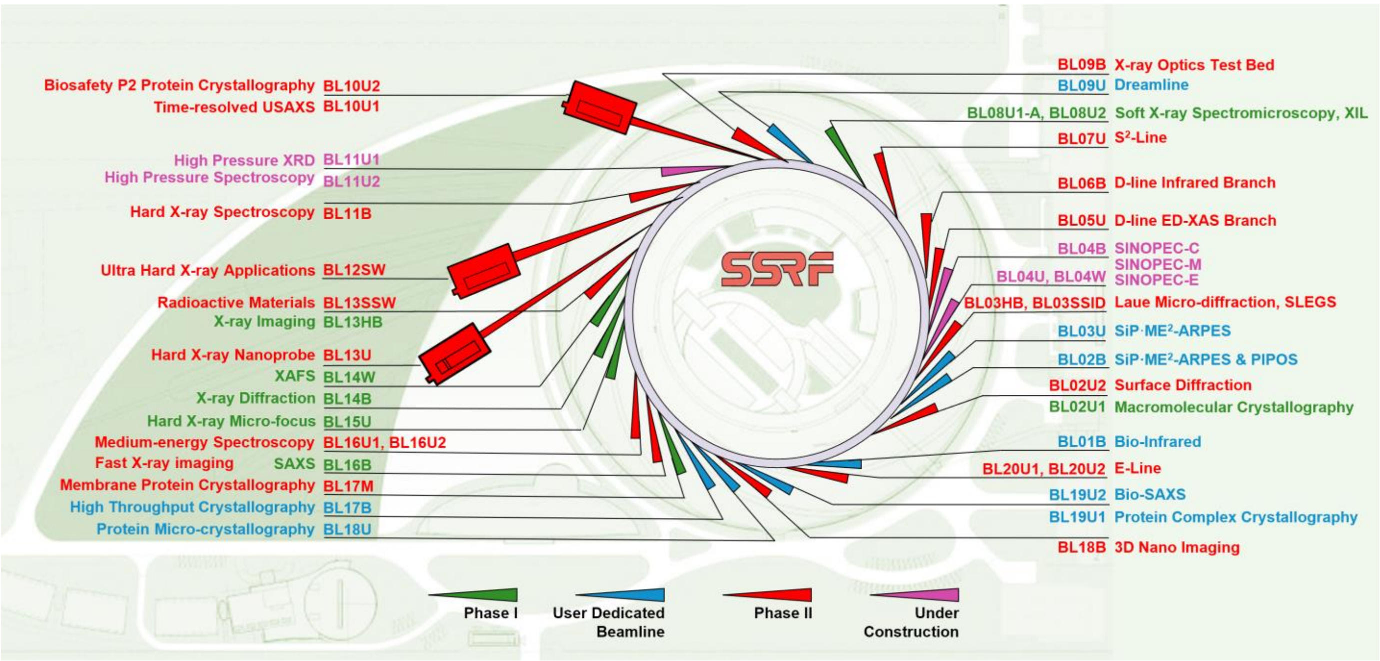

Figure S1 SSRF beamline layout.

Table S1 Functional Modules of the SR-CT framework.

Category	Module	Function	Deployed at
Pipeline	Tomography Linux Pipeline API	API for submitting pipeline tasks to the BDSC and checking pipeline status	BDSC clusters
	GPU Pipeline Driver	Ensure seamless task submission to the BDSC clusters	
	Pipeline Message Driver	Driver translating the pipeline messages sent to Kafka	
Service	Tomography Metadata API	API for importing and cataloguing metadata	BDSC supporting servers
	Tomography Message Consumer	Receive pipeline messages from Kafka and initiate the metadata collection in real-time	
	Tomography Collector Timer	Governing the metadata collection, based on time rules	
	Tomography Metadata Core Service Modules	Integrate and manage the parsing, loading, transferring and mapping of the sources to the metadata format, and import them into the SSRF-SciCat	
	Tomography Metadata Transforming and Mapping	Finalize the transformation and mapping of the sources to the SSRF-SciCat metadata.	
	HPC Metadata Extract-Transform-Load (ETL)	The architectural framework module that can connect, extract, transfer and load the metadata from the HPC scheduler	
	HPC Metadata Driver	Parsing the metadata from the BDSC HPC cluster	
	Imaging Parameter Collector	The framework module that can collect and parse the metadata from the imaging application parameter files	

	Parameter Driver	Parser for the metadata from the imaging application parameter files	
	Files ETL	The architectural framework module that can connect, extract, transfer and load metadata from the storage	
	Files Driver	Parser for the metadata from the file storage	
Client	Tomography Windows/Linux API	Windows/Linux API for the imaging application	Beamline workstations
	Tomography Input Data Parser	Parse the parameters from the input files generated by the imaging application, and created new parameter files for the BDSC pipeline	
	Tomography File Synchronizer	Automatically synchronize the local files to the BDSC storage	
	Tomography Windows/Linux Pipeline Driver	Submit remote task on Windows and Linux	
Environment	SSRF AD Domain	Provides account authentication for the clusters and beamlines	BDSC AD servers
	BDSC Virtualization and Storage	Makes the BDSC storage accessible to the beamlines	BDSC storage servers
	Kafka Cluster	Provides the remote message services	BDSC supporting servers
	Zookeeper Health Cluster	Provides Kafka and nodes coordination	BDSC supporting servers
	SSRF-SciCat	Integrate the SciCat core components into the BDSC scientific computing framework	BDSC supporting servers
	MongoDB	Metadata database	BDSC supporting servers
	Containers	Containerization services	BDSC supporting servers

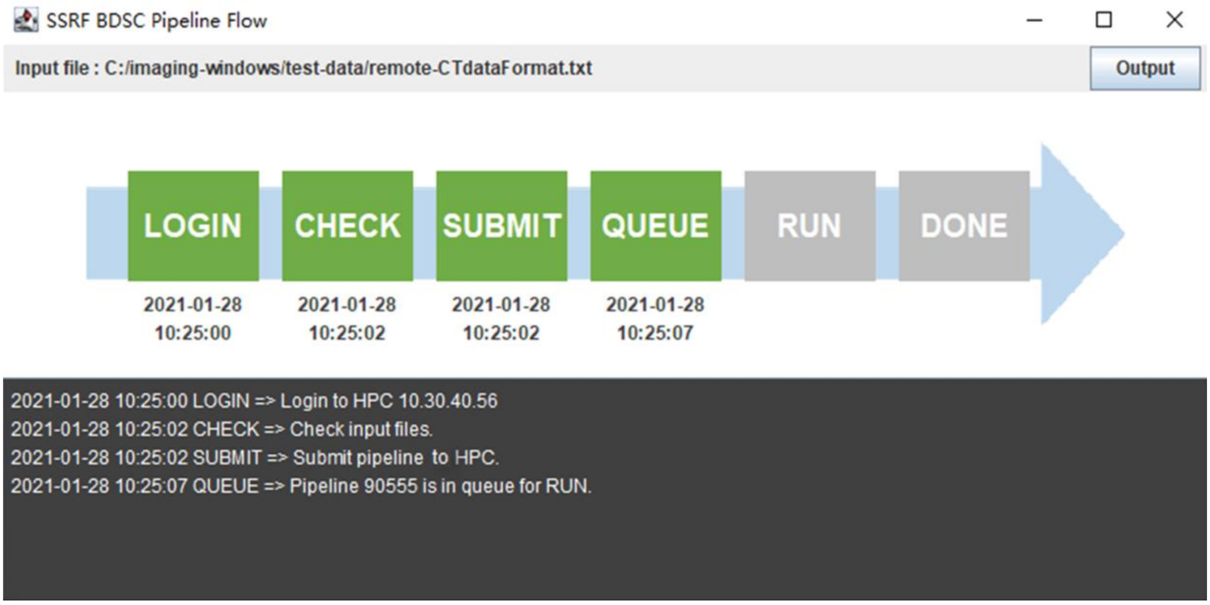

Figure S2 GUI of the SR-CT pipeline framework. Each step is executed with high time accuracy (seconds).
